# Filtering "genic" open reading frames from genomic DNA samples for advanced annotation

**DOI:** 10.1186/1471-2164-12-S1-S5

**Published:** 2011-06-15

**Authors:** Sara D'Angelo, Nileena Velappan, Flavio Mignone, Claudio Santoro, Daniele Sblattero, Csaba Kiss, Andrew RM Bradbury

**Affiliations:** 1 Bioscience Division, Los Alamos National Laboratory, Los Alamos, NM, USA; 2 Department of Structural Chemistry and Inorganic Stereochemistry, School of Pharmacy, University of Milan, Milan, Italy; 3 Department of Medical Sciences and IRCAD, University of Eastern Piedmont, Novara, Italy

## Abstract

**Background:**

In order to carry out experimental gene annotation, DNA encoding open reading frames (ORFs) derived from real genes (termed "genic") in the correct frame is required. When genes are correctly assigned, isolation of genic DNA for functional annotation can be carried out by PCR. However, not all genes are correctly assigned, and even when correctly assigned, gene products are often incorrectly folded when expressed in heterologous hosts. This is a problem that can sometimes be overcome by the expression of protein fragments encoding domains, rather than full-length proteins. One possible method to isolate DNA encoding such domains would to "filter" complex DNA (cDNA libraries, genomic and metagenomic DNA) for gene fragments that confer a selectable phenotype relying on correct folding, with all such domains present in a complex DNA sample, termed the “domainome”.

**Results:**

In this paper we discuss the preparation of diverse genic ORF libraries from randomly fragmented genomic DNA using ß-lactamase to filter out the open reading frames. By cloning DNA fragments between leader sequences and the mature ß-lactamase gene, colonies can be selected for resistance to ampicillin, conferred by correct folding of the lactamase gene. Our experiments demonstrate that the majority of surviving colonies contain genic open reading frames, suggesting that ß-lactamase is acting as a selectable folding reporter. Furthermore, different leaders (Sec, TAT and SRP), normally translocating different protein classes, filter different genic fragment subsets, indicating that their use increases the fraction of the “domainone” that is accessible.

**Conclusions:**

The availability of ORF libraries, obtained with the filtering method described here, combined with screening methods such as phage display and protein-protein interaction studies, or with protein structure determination projects, can lead to the identification and structural determination of functional genic ORFs. ORF libraries represent, moreover, a useful tool to proceed towards high-throughput functional annotation of newly sequenced genomes.

## Background

Advances in sequencing technologies have led to the explosion of large-scale sequencing projects: as of January 2011, 1331 bacterial genomes have been successfully sequenced, with other 4424 genomes either unfinished or in assembly phases (http://www.ncbi.nlm.nih.gov/genomes/lproks.cgi). Since sequencing is no longer an issue, the real challenge is understanding how DNA sequence leads to the specific phenotype of an organism. A key step in this process is the annotation of genes encoding proteins that contribute to particular functions. Since the completion of the first bacterial genome in 1998, this process is usually carried out automatically using *ab initio*, homology, or combination approaches [1]. Most gene structures are based on computational predictions [2], and annotation is based on homology. However, automated annotation can be incorrect when sequence similarity is not associated with functional similarity, or when reference databases contain incorrect annotations, a problem that is estimated to afflict up to 49% of genes in public databases [3-6]. Gene function is originally assigned where there is homology with related genes whose activity has been determined experimentally. However, second and third generation annotations, as well as associated errors, are prevalent.

Experimental information related to protein function is necessary, but far more difficult to obtain at a whole genome level. Genes must be informatically identified before they can be cloned, expressed, and tested for function, and the study of a whole recombinant proteome relies on the cloning of all Open Reading Frames (ORFs) in a genome. These “ORFeome” collections, as they are termed, require huge efforts in terms of time and resources. Once cloned, recombinatorial cloning systems allow relatively straightforward transfer between different vectors for genome scale projects [7]. However, even after correct gene identification and cloning, challenges are still present in terms of full-length protein expression and purification, with as few as 30% of proteins expressed solubly in *E. coli* at sufficient levels to be experimentally useful [8,9].

Functional annotation would be greatly facilitated if genomic DNA could be used directly without the need to create ORFeome resources. The fact that proteins generally contain multiple domains, each of which contributes to a distinct function, provides a potential mechanism by which this can be carried out. Once generated, a protein domain library will be useful for many purposes. Applications such as structural studies, antibody generation, protein/substrate binding analyses, domain shuffling for enzyme evolution and protein chips will all benefit from a library of well folded protein domains.

An analysis of the length distribution of protein domains [10], reveals that most range from 50 to 200 aa, with a peak at around 100 aa: we speculate that fragmenting a whole (intronless) genome into DNA fragments of 200-800 bp, should provide a broad representation of all the protein domains from a single species, a polypeptide population that has been termed the “domainome” [11]. The availability of genome sequences, coupled with the ability to experimentally determine the function of a domain, rather than a full-length protein, could also provide a simpler method to annotate genes on the basis of specific function.

In order to develop a gene annotation method based on the function of individual domains on a genomic scale, a random approach is required in order to avoid bias towards what is already known. In this perspective, randomly fragmented genomic DNA represents a good DNA source for intronless organisms. Unfortunately, the use of randomly generated DNA fragments suffers from several problems: i) non ORFs (ORFs containing suppressible stop codons) and non genic ORFs (alternative ORFs in a frame other than the original frame of the gene (genic ORFs)), can be obtained for fragments derived from random fragmentation. These non genic ORFs will translate into polypeptides with no biological meaning; ii) folding failure occurs even for correctly identified and cloned ORFs, thus impairing their function; iii) proteins or protein fragments which fold in different cellular compartments can be affected by recombinant expression in inappropriate redox, or chaperone, environments.

To address these issues, we demonstrate [12-14], with others [15-17], that folding reporters could be very useful tools. The principle under which they operate is that a poorly folded test protein can adversely affect the folding of a “reporter” protein to which it is fused, by trapping it in a non-functional or aggregated state. Moreover, when the folding reporter has an easily identifiable phenotype (e.g. antibiotic resistance [12], fluorescence [15], or color complementation [16]), rescuing those clones expressing properly folded and soluble ORFs becomes a relatively straightforward process.

While it might be expected that any ORF (genic or non genic) would confer the positively selectable phenotype (fluorescence, antibiotic resistance), we recently observed that when cDNA fragments are cloned upstream of the folding reporter, a selection for fragments from real genes tends to occur [18], and when a plasmid containing four known genes was fragmented and placed upstream of a folding reporter, over 80% of selected DNA fragments were genic ORFs [12].

**Figure 1:**
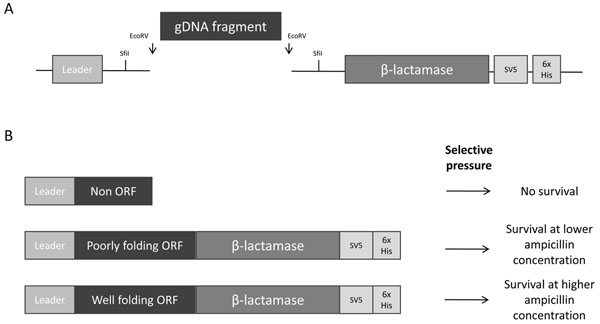
**ORF filtering vector.** The filtering vectors features are shown in panel A. Blunt ended gDNA random fragments are cloned between a leader sequence (Sec, SRP, or TAT) and the mature β-lactamase gene. C-terminal SV5 and His tags are used for detection and purification respectively. In panel B, the effect of selective pressure on ampicillin is shown for ORFs and non ORFs.

The folding reporter we used in this work is the TEM-1 β-lactamase. This enzyme confers ampicillin resistance to bacteria only when exported into the periplasmic space, a process that relies on the presence of a secretion leader at the N-terminus of the enzyme. In our system, DNA fragments are cloned between the secretion leader and the β-lactamase gene (Figure 1), so that only those fragments that are able to fold coherently, and are in frame with both components, allow correct folding of the enzyme and its subsequent export into the periplasm. Moreover, the double fusion ensures that small fragments containing cryptic start sites cannot be positively selected, as they lack the leader sequence, resulting in an increase in the stringency of selection. This is in contrast to other folding reporter models [15,19], in which fragments are cloned upstream of the reporter gene alone. As M13 phages assemble in the periplasm, a protein domain library translocated into the periplasm is also suitable for M13 based phage display, providing appropriate vectors [20] are available. This allows domainome libraries to be directly selected for gene fragments encoding domains with specific binding properties or even enzyme activities, if compatible activity based probes [21] are available.

Proper folding of a polypeptide depends both on the amino acid composition and extrinsic factors, such as chaperones and redox conditions. Consequently correct folding depends upon a protein folding in the appropriate cellular compartment.

In bacteria, most proteins cross the inner membrane by means of the type II secretory system. This comprises three different pathways [22]: the Sec-dependent pathway , the signal recognition particle (SRP), and the twin-arginine translocation (TAT) pathways. While the Sec pathway exports unfolded proteins, the SRP and TAT pathways export co- and post-translationally folded proteins, respectively. In this paper, we show how the use of three different β-lactamase filtering vectors, each exploiting one of the different export systems, allows broader representation of domains that can be filtered from a genome.

In order to demonstrate the feasibility of the filtering method applied at the whole genome level, we chose *Clostridium thermocellum* as model organism. The general interest in this anaerobic bacterium relies on its extraordinary ability to metabolize plant cell wall polysaccharides by means of a complex secreted multiprotein complex. Its components have a typical multidomain structure where each domain has a defined function (e.g. anchoring onto substrate, anchoring on bacterial membrane, adaptor domains, catalytic domains) [23]. Indeed, the fact that its genome has been recently sequenced (GenBank ID: CP000568.1), but not completely annotated makes it a good candidate for domain-based functional annotation purposes.

In this paper we describe the preparation and characteristics of filtered domain libraries prepared from genomic DNA, using libraries created from the *C. thermocellum* genome as a model system.

## Results

### Construction of *C. thermocellum* DNA libraries and ORF filtering

Due to the relatively high amount of starting material required to generate a genomic library, *C. thermocellum* genomic DNA (gDNA) was used as a template for multiple displacement amplification (MDA) in order to obtain 10-20 µg of DNA for subsequent fragmentation. Fragmentation was carried out using nebulisation, and conditions were optimized to obtain a fragment distribution in the range of 200-800 bp. Such a length range was intended to be optimal for the statistical representation of all the domains in the genome. gDNA was cloned as blunt end fragments into three filtering vectors, in which an EcoRV cloning site was found between a Sec (POS), SRP (SOS) or TAT (TOS) leader sequence and the mature ß lactamase. The three final vectors, carrying the chloramphenicol resistance gene as a selective marker, have the SV5 and 6xHis tags downstream of the cloned gDNA fragment (see Figure 1, panel A). The effect of growing clones containing the gDNA library on different concentrations of ampicillin is depicted in Figure 1, panel B: only gDNA fragments that are in frame with both secretion leader and β-lactamase gene (ORFs) can survive the selective pressure of higher ampicillin concentrations. Among surviving clones, those that encode well-folding fragments would be expected to survive at higher ampicillin concentrations, as they would be expected to allow greater amounts of functional β-lactamase to accumulate.

Fragment length was provisionally assessed by PCR analysis of random clones, and the average length determined to be around 400 bp. Considering the starting diversity of the non-filtered libraries (4x10^6^ for SOS and 1x10^7^ for POS and TOS), a statistical coverage of 400 to 1000 fold of the 3.8 Mb genome was obtained. With such coverage, we expected that, after ORF filtration the final library diversity would have remained sufficient to represent all *C. thermocellum* genes.

**Figure 2:**
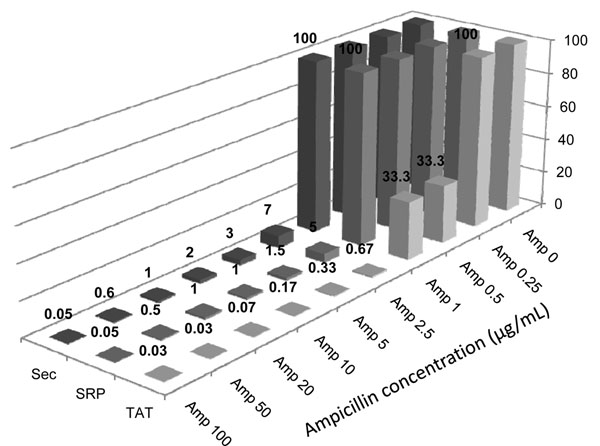
**Filtering of *Clostridium thermocellum* gDNA libraries.** Survival rates of the three (Sec, SRP, TAT) genomic DNA fragmented libraries plated on increasing ampicillin concentrations are shown. Data are normalized according to the total number of clones growing on Chloramphenicol (Amp 0) plates, with no filtering pressure, and indicated as percentages.

Approximately 10^8^ bacterial cells for each library were plated on chloramphenicol plates, supplemented with increasing ampicillin concentrations (ranging from 0 to 100 µg/mL). After overnight growth at 30°C, the number of surviving cells was evaluated with data shown in Figure 2. The Sec and SRP libraries show similar behaviour, with a dramatic drop off at 2.5 µg/mL ampicillin, while the TAT library has a more gentle decrease in survival rate, with a first drop at 0.5 µg/mL, and a second one at 2.5 µg/mL, when survival seems comparable to the other two libraries. The ampicillin concentration at which 1% of clones survive is different for the three libraries. The Sec leader provides the greatest resistance, requiring ~20 µg/ml, the SRP leader requires 10 µg/ml, and the TAT leader is the most sensitive, with 2.5 µg/ml ampicillin being sufficient to eliminate over 99 % of colonies.

### β-lactamase functional assay on filtered libraries

In order to confirm the hypothesis that some colonies can survive at higher ampicillin concentration because a greater amount of soluble and functional β-lactamase fusion protein is produced, we tested the β-lactamase activity of random clones grown on agar plates containing different concentrations of ampicillin.

**Figure 3:**
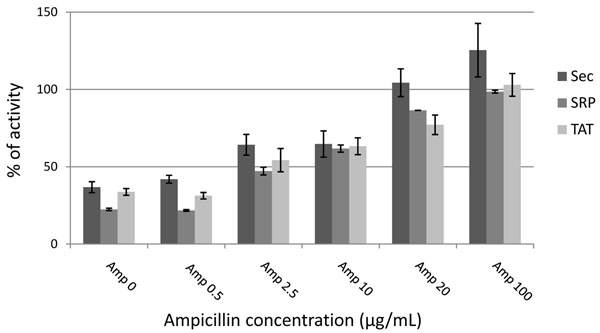
**β-lactamase assay on non filtered and filtered libraries.** In the chart, the mean activity value of 45-48 clones for each ampicillin concentration in 3 replicate plates is represented. Data collected at 6 h incubation time-point for Sec, SRP, and TAT libraries are shown.

Forty-five to 48 clones for each library were picked from chloramphenicol plates (representing unfiltered clones) and from plates with 0.5, 2.5, 10, 20, and 100 µg/mL ampicillin; culture supernatants of induced clones were tested for activity on nitrocefin, a chromogenic substrate containing a β-lactam ring that is hydrolyzed by β-lactamase. The activity was measured as an increase in the absorbance at different time-points; positive and negative controls were also included. As shown in Figure 3, the average activity of clones in each of the three libraries increased with the increasing selective pressure provided by increasing ampicillin concentrations. Additional file 1 shows the clones tested for the TAT library. Pictures of plates (panel A) and corresponding absorbance measurements (panel B) were taken at 2, 6, and 16 h. The data show that most of the clones surviving at 2.5 µg/mL and higher ampicillin concentrations are actually ORFs, since all of them have β-lactamase activity at 16 h; interestingly, clones surviving at higher ampicillin concentrations (20 and 100 µg/mL) already have higher activity at 2 h incubation, while signals from clones surviving at 0.5-10 µg/mL rise more slowly. This is an indication that the activity is enhanced for clones surviving at higher ampicillin concentration: this finding is best interpreted as indicating that a higher expression of correctly folded, active and soluble fusion enzyme is produced at the higher ampicillin concentrations.

### Deep sequencing of filtered libraries

**Figure 4:**
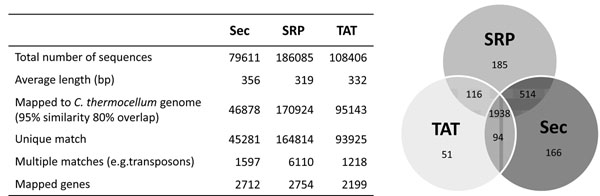
**454 sequencing analysis of filtered libraries.** Sequencing data for the 3 libraries Sec, SRP, TAT) are shown in the table. The Venn diagram shows the number of different genes shared between the libraries.

**Figure 5:**
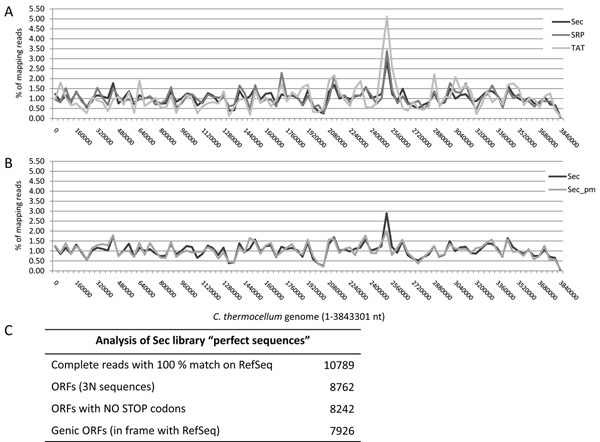
**Sequence distribution along *C. thermocellum* genome.** Panel A shows the distribution frequencies of 454 sequences along *C. thermocellum* genome in 40000 nucleotides windows. Panel B shows the distribution of Sec filtered libraries compared with the distribution of the perfect match (pm) sub set of sequences from the same library. Panel C shows the 454 data analysis for perfect match sequences in Sec library.

Surviving bacteria were rescued from the different filtering plates and plasmid DNA obtained for each sample. We chose to sequence the samples with a survival rate of approximately 1%, reasoning that this should identify clones encoding domains that are relatively well folded. Inserts from filtered libraries were cleaved by restriction enzymes and subjected to Roche 454 FLX titanium sequencing. Sequences were aligned to the reference *C. thermocellum* genome and 96% (3064 of 3189 genes) of annotated protein coding genes were identified by at least one read (see Table in Figure 4). The Venn diagram in Figure 4 shows the distribution of the mapped genes, with a significant portion of genes represented in only one of the libraries, thus sustaining the hypothesis that, by using 3 vectors with different leaders, domain representation is increased. Figure 5, panel A, shows the general distribution of reads along *C. thermocellum* genome, indicating approximate even representation of the entire genome, with only one region showing a significant over-representation. Furthermore, careful examination of figure 5A reveals a number of regions where the three libraries show different percentage representations. Interestingly, the Sec library gave the highest coverage, despite the lower number of sequences obtained. A total of 79611 reads were obtained for the Sec filtered library, with 2712 genes mapped by at least one read.

As 454 sequencing normally introduces errors [24], it was impossible to accurately determine the percentages of genic or non genic ORFs that were filtered. In order to overcome this, we increased the stringency of the sequence analysis by creating a data set of “perfect sequences” with no sequencing errors and 100% match to the genome reference sequence (RefSeq). This procedure led to the identification of a data set of 10789 perfect sequences, with the same distribution pattern as the general Sec library (see Figure 5, panel B), thus indicating that no bias was introduced when increasing the stringency of the sequence analysis. Within the “perfect match” set of sequences, the reading frame could be correctly and unambiguously assigned (see Figure 5, panel C for more details): 73.5 % of reads corresponded to genic open reading frames, a much higher percentage than the expected 17 % (1 fragment out of 6 possible frames is expected to be in the same frame of the corresponding gene by chance). These data indicate that β-lactamase acts as a folding reporter, thus pushing the selection towards ORFs with biological meaning.

**Figure 6:**
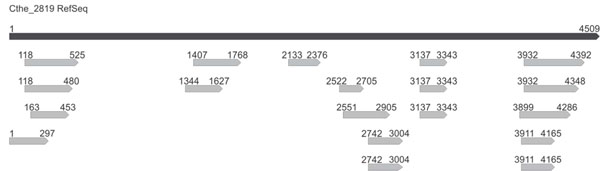
**Filtered fragments distribution on single genes.** The distribution and the variable length of Sec filtered clones mapping on Cthe_2819 gene are shown. The mapping reads were obtained from the raw 454 sequencing dataset.

A more detailed analysis of a number of genes covered by multiple clones showed that sequences with different lengths were evenly distributed along the genes. In Figure 6, an example is given for filtered sequences mapping to Cthe_2819. As can be seen, much of the gene is covered by sequences, and some of these overlap in what appear to be potential domains. The true coverage is likely to be significantly higher, since we were only able to sequence a small proportion of the complete filtered library size.

## Discussion

In a previous paper [12] we fragmented a plasmid containing four genes (and 62 non-genic open reading frames greater than 150bp), and used the ß lactamase approach described here to “filter” out putative open reading frames. We discovered that all filtered clones were open reading frames and 84% of these were derived from real genes, as opposed to random open reading frames. In the work described here, we have extended these results to the analysis of a full genome. This was carried out in two parts. In the first part the genome coverage for each of the three libraries (Sec, SRP and TAT leaders) was assessed using the obtained sequences. The results in Figure 4 showed that almost all of the annotated genes in *C. thermocellum* were represented by at least one read in the library. Furthermore, the use of different leaders increased the genome coverage. Most genes (1938, or 61%) were found in all three libraries. The Sec leader provided the greatest number of different genes (2712 genes or 85% of the total), and the addition of the TAT and SRP leaders provided an additional 352 (11%) different genes, for a total of 96% of all genes, or almost complete genome coverage. The goal of the second analysis was to determine the percentage of open reading frames, and where they were derived from. Given the tendency of 454 sequencing to introduce errors [24], it was impossible to carry this out with the raw 454 sequences. This was overcome by compiling a set of “perfect sequences” that had a 100% match to the genome sequence at both ends, allowing us to determine the precise start and end of each clone. Perfect sequence sets were generated from the Sec library filtered to a survival level of 1%. These revealed that 76.4 % were open reading frames with no stop codons, of which 96.2 % were derived from genic ORFs, as opposed to spurious ORFs, of no biological significance. We hypothesize that real gene or mRNA fragments encode polypeptides that naturally evolved to fold correctly, thus driving the proper folding and activity of the folding reporter, while random ORFs generate peptides with no biological meaning that are more likely to negatively affect the folding, aggregation state or activity of the reporter.

In our sequencing experiments, we analyzed those clones corresponding to a survival of 1%. We reasoned that this would represent a balance between broad genome representation and the selection for clones encoding well folding domains. Figure 3 and additional file 1 show clearly that the greater the concentration of ampicillin used for filtering, the higher the activity of ß-lactamase, as determined by the nitrocefin colorometric assay. Although we have not formally shown that domains fused to ß-lactamase with higher activity are better folded, similar experiments using GFP (green fluorescent protein) as a folding reporter [15,25], in which proteins of interest are fused upstream of GFP and selected on the basis of clone fluorescence, have clearly demonstrated that clone fluorescence is directly proportional to the folding and solubility of the fused protein of interest when not fused to GFP. In these experiments, GFP can be considered to be analogous to the ß-lactamase, in that correct functioning of the reporter is dependent upon the folding and solubility state of the fused domain. The technology described here is genome neutral and can be used to rapidly create a domainome library from any intronless genome, or collection of open reading frames of interest. The use of the random approach described here avoids the need for extensive analysis, primer synthesis or multiple PCRs, and creates a resource in which many different versions of each domain, differing by a few amino acids, are created. Once generated, it is expected that the protein fragments obtained by this approach will be useful for many purposes, including structural studies, antibody generation, protein/substrate binding analyses, domain shuffling for enzyme evolution and protein chips. Furthermore, once recloned into a phage display context, domainome libraries can be directly selected for gene fragments encoding domains with specific binding properties (e.g. to other proteins, domains, enzyme substrates) or enzyme activities, if appropriate activity based probes are available [21]^21^[21][20]^20^[20].

## Conclusions

With this work we demonstrated that domainome libraries can be easily generated by applying β-lactamase based filtering to randomly fragmented bacterial gDNA libraries. Once a library is generated, it can be used as a universal reagent to be screened for several activities. The identification of domains showing specific activity, instead of the testing of single genes, will allow functional annotation of the domains themselves: this annotation represents the first step to the high throughput assignment of full length gene products to structural functions or to specific metabolic pathways.

## Methods

### Genomic DNA library construction

Genomic DNA from *Clostridium thermocellum* (ATCC 27405) was kindly provided by Prof David Wu, Univ. Rochester. DNA was amplified by multiple displacement amplification (MDA) with a Repli-g screening kit (Qiagen) according to the manufacturer’s instructions. After 16 h amplification, DNA was fragmented using nitrogen gas based nebulisation for 1 min at 45 psi; average fragment size was around 500 bp (200-800 bp range).

Fragments were twice blunt-ended (Quick blunt kit, NEB) and gel-purified (Gel extraction kit, Qiagen) before cloning into the EcoRV cleaved POS, SOS, and TOS vectors. These vectors were obtained from the original pPAO phagemid vector [12], by removing the g3p gene and either maintaining the Sec secretion leader (in POS vector) or replacing it with SRP and TAT leaders (in SOS and TOS respectively) encoded by oligonucleotides.

After ligation, the three libraries were electroporated into *E. coli* DH5α F’ cells, plated on chloramphenicol agar plates and grown for 16 h at 37°C.

### ORF filtering

The genomic DNA fragmented libraries obtained in the three vectors were harvested from plates; 10 µL of each library were diluted in LB media to OD_600_ 0.5 and 100 µL (10^8^ cells, corresponding to 10-20 fold the starting library diversity) were plated on plates supplemented with chloramphenicol (34 µg/mL) alone, or containing ampicillin at different concentrations, ranging from 0.25 to 100 µg/mL. Plates were incubated for 20 h at 30°C.

### 454 sequencing and data analysis

Each library filtered to a survival rate of around 1% underwent deep sequencing. Filtered gDNA was removed as SfiI oriented fragments from the purified plasmid DNA. One µg of gel extracted DNA fragments for each library was used as starting material for the preparation of the 454 tagged libraries. DNA quality control was performed using the Qubit HS quantitation platform (Invitrogen); ligation of purified samples to specific adaptors and preparation of the single strand libraries (ssDNA) were performed following the manufacturer’s instructions (GS-FLX Titanium kit, Roche). The quality control on the ssDNA libraries was performed by capillary electrophoresis (Agilent Bioanalyzer 2100 with the RNA Pico 6000 LabChip kit; Agilent Technologies). The ssDNA libraries were then processed as required by the 454 sequencing protocol. Each enriched sample was separately loaded onto one-eighth of the PicoTiterPlate and sequenced.

Raw data were processed by a custom-made workflow procedure mainly based on PERL scripts. Briefly, sequences were mapped onto the *C. thermocellum* genome (Reference Sequence CP000568.1) using Gmap software [26] and matching sequences were compared with annotated genes. Each gene was then identified by the number of mapping reads; a further implemented reiterated procedure allowed us to analyze each library in terms of mapping, annotation and filtering features. Data are accessible through a website interface implemented in php and java (http://www.interactomeataglance.org). See additional information for browsing directions.

### β-lactamase functional assay

48 clones for each library were picked from plates at different ampicillin concentrations (ranging from 0 to 100 µg/mL). After O/N growth in autoinduction media [27], culture supernatants were collected and tested for β-lactamase activity in a nitrocefin-based functional assay. Briefly, nitrocefin (EMD, Calbiochem) was diluted to 100 µg/mL in PBS and 50 µL of this working solution were added to 10 µL of cultures supernatant. The assay was performed in the 96-well plate format and plates were read at 486 nm wavelength with a microplate reader (Infinite M200, Tecan) at different time-points (2 h, 6 h, 16 h). Signals were normalized to the positive control signal per each plate; the β-lactamase activity for each clone was reported as a percentage value (where the positive control has 100 % activity).

## Competing interests

The authors declare that they have no competing interests.

## Authors' contributions

SDA and NV performed the experiments; SDA wrote the manuscript. FM developed and applied the informatic analysis tools. CS and DS contributed to the development of the filtering method and provided scientific support; CK helped supervise the experiments and edit the manuscript. ARMB conceived the filtering method, supervised the experiments, was responsible for obtaining funding, and edited the final manuscript draft.

## Appendix

### http://www.interactomeataglance.org website access and navigation

This website provides access to the data for this and previous works published by our group.

The *Clostridium thermocellum* project link is accessible from the home page. The page briefly describes the project and, on the top of the blue ribbon, the “Browse data” button allows access to the “Cthe_library” dataset. When clicking on it, a new page opens, with the list of the available sub-sets and their brief general description at the bottom of the page (the description includes total number N# of reads, average length, number N# of mapping reads and number N# of genes mapped).

Four datasets, (labeled as Sec, Sec_pm, SRP, TAT) are available on the “Select” page: by checking one or more of the corresponding boxes, the dataset/datasets of interest can be browsed.

After the selection of the datasets, 2 browsing options are available after clicking on “Filter data”:.

- “Query Gene” button: this allows the database to be interrogated for specific genes. Enter the HUGO ID (e.g. Cthe_2819) and press the “Submit Query” button. A new window appears: the gene of interest is characterized by “Coverage” (N# of sequences mapping on the gene), “Depth” (N# of overlapping sequences on the gene), “Focus” (index of the overlapping: 1 means 100 % sequences sharing a common region, 0 means sequences spread along the gene, with no overlapping). The “Known introns” and “New introns” labels are not relevant to this project; the “Flag” label indicates the dataset to which the previous features (Coverage, Depth, Focus) refer. Clicking on the “eg_ID” redirects on the actual browser, where sequences are shown as segments mapping on the genomic sequence of *C.thermocellum*. Blue arrows indicate the annotated gene and red bars indicate the RefSeqs. Color coded bars (from green to blue) indicate the number of sequences mapping on the specific region in the datasets (see labels on the left). The region shown can be modified either by indicating specific genomic coordinates on the top “Displayed region” bar and clicking on the “Refresh” button or by clicking on the”+” and “-“ bottons”. Alternatively, it is possible to center and magnify the selection on a specific region by drawing a window on the genic region of interest and clicking on the “Center on Selection” button.

Among the several other browsing options available on the “Features to display” bar, the “Reads” window allows to visualize the actual reads mapping on the specific region (select the “Full” option and “Refresh”).

The actual nucleotide sequences of the specific genomic region and of the mapping reads can be downloaded by drawing a window on the browser and clicking on the “Download sequences” button.

- “Filter data” button: the “Advanced search” button allows the selection of only those genes showing desired features (sequence depth and/or coverage, focus index). For example, in order to visualize all genes mapped by at least 1 read in the Sec dataset, check the “Sec” box, select the “>=” option and enter “1” in the “Min. sequencing coverage” lane. The table provided shows the genes matching the filtering parameters previously defined. The genes can be ranked by the header selected (“hugo”, “coverage”, “depth”, and “focus”) and can be browsed afterwards by clicking on the “eg_ID” number.

## Supplementary Material

Additional file 1 **β-lactamase assay on TAT library.** Panel A shows 45-48 TAT clones for each filtering concentration in the 96 well format nitrocefin (NC) assay. Clones correctly expressing a functional β-lactamase fusion in the supernatant turn from yellow to red. Controls are shown in black circles. In panel B, absorbance measurements performed at 2 h, 6 h, and 16 h (saturation point) are shown as average measurement of triplicate plates. Data were normalized on the positive control signal (shown as black bar with 100% signal) and reported as percentage value. Click here for file
