# Supplementary figures and images for: Filtering "genic" open reading frames from genomic DNA samples for advanced annotation
Source: BMC Genomics. 2011 Jun 15;12(Suppl 1):S5. doi: 10.1186/1471-2164-12-S1-S5 (PMC3223728; doi:10.1186/1471-2164-12-S1-S5)

Supplementary Figure 1 -  $\beta$ -lactamase assay on non filtered and filtered libraries

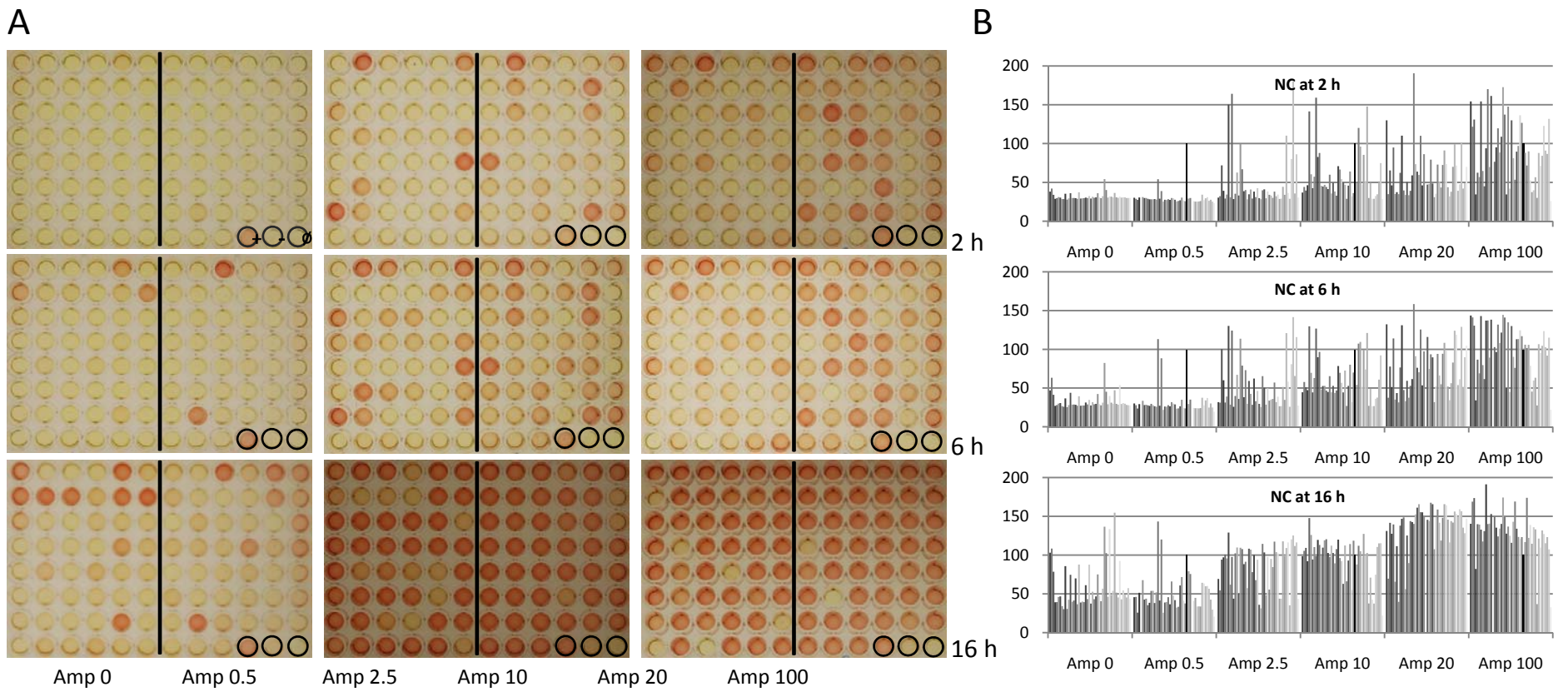

Supplement: Additional file 1 — β-lactamase assay on TAT library. Panel A shows 45-48 TAT clones for each filtering concentration in the 96 well format nitrocefin (NC) assay. Clones correctly expressing a functional β-lactamase fusion in the supernatant turn from yellow to red. Controls are shown in black circles. In panel B, absorbance measurements performed at 2 h, 6 h, and 16 h (saturation point) are shown as average measurement of triplicate plates. Data were normalized on the positive control signal (shown as black bar with 100% signal) and reported as percentage value. [file 1471-2164-12-S1-S5-S1.pdf]
